# Editorial: Insights in intensive care cardiovascular medicine: 2022

**DOI:** 10.3389/fcvm.2024.1446904

**Published:** 2024-08-06

**Authors:** Fabio Guarracino, Marija Vavlukis

**Affiliations:** ^1^ Department of Anaesthesia and Intensive Care Medicine, Azienda Ospedaliero Universitaria Pisana, Pisa, Italy; ^2^ Azienda Ospedaliero Universitaria Pisana, Pisa, Italy; ^3^ Faculty of Medicine, University Clinic for Cardiology, University Ss’ Cyril and Methodius in Skopje, Skopje, North Macedonia

**Keywords:** shock, cardiogenic, organ dysfunction, multidisciplinary approach, pharmacological approach

**Editorial on the Research Topic**
Insights in intensive care cardiovascular medicine: 2022

**Figure 1:**
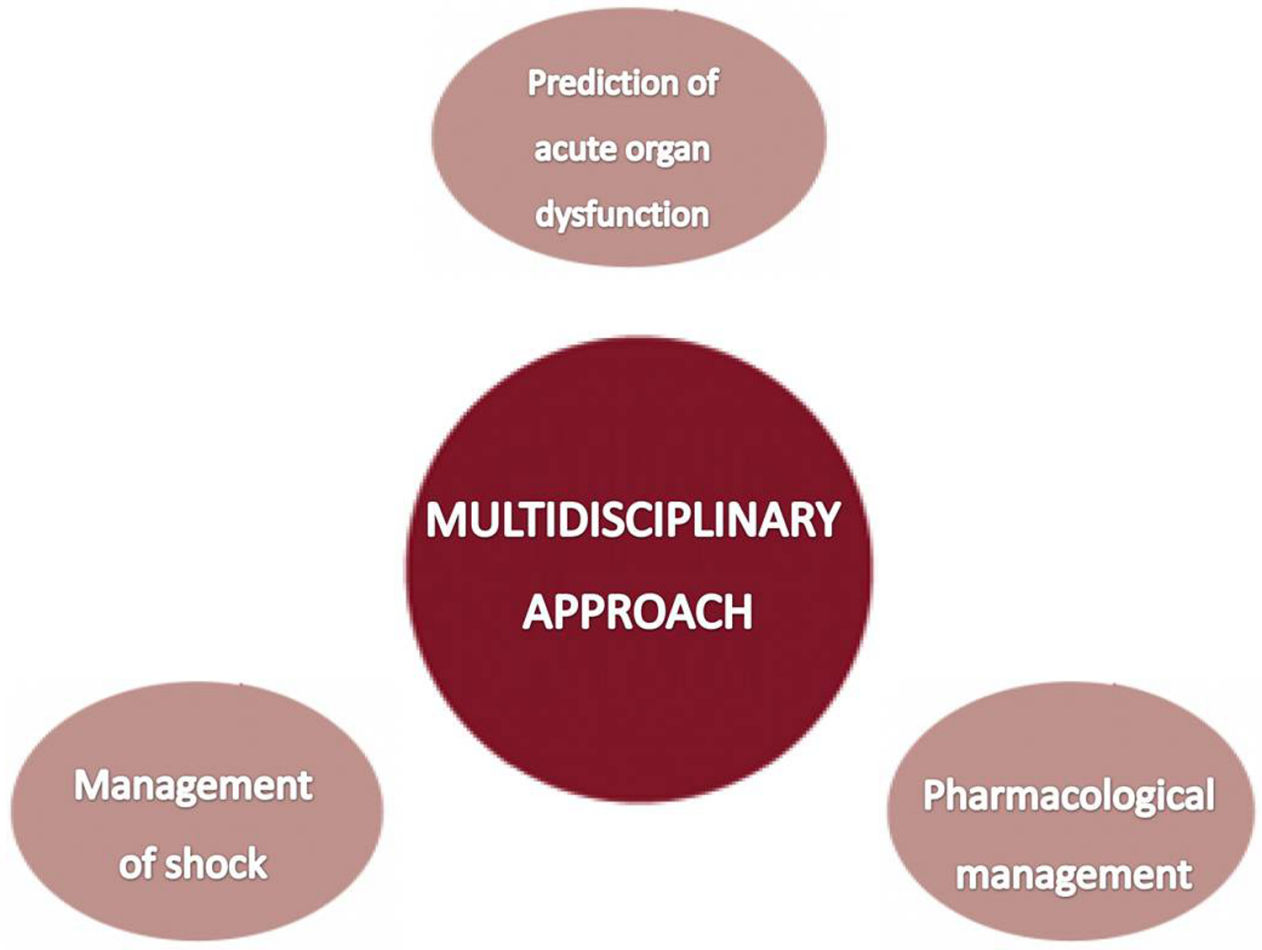
The major areas covered by the Research Topic “*Insights in Intensive Care Cardiovascular Medicine: 2022*”.

The Research Topic “*Insights in Intensive Care Cardiovascular Medicine: 2022*” included nine manuscripts addressing four major areas in the field (Figure 1): the multidisciplinary approach to treating cardiovascular issues, the challenges of cardiogenic shock, prediction approaches to organs' acute dysfunction, and the pharmacological management of acute cardiovascular conditions.

The need for a multidisciplinary approach in the clinical management of acute cardiovascular states was explored in an interesting contribution to the Research Topic (Bouchlarhem et al.). It clearly underlined that the field of cardiac intensive care has evolved significantly since Desmond Julian's establishment of the first coronary intensive care unit (CICU) in 1961. Originally designed to improve the prognosis of myocardial infarction patients, CICUs have since expanded to address a wide range of acute cardiovascular conditions like severe arrhythmias, acute heart failure, cardiogenic shock, high-risk pulmonary embolisms, severe conduction disorders, post-implantation monitoring of percutaneous valves, and non-cardiac emergencies like septic shock, severe respiratory failure, and severe renal failure, along with managing cardiac arrest post-resuscitation. This evolution has included the incorporation of advanced therapeutic techniques such as fibrinolysis, invasive hemodynamic monitoring, and mechanical circulatory support, along with percutaneous coronary and structural interventions. Consequently, the transition to more comprehensive cardiac intensive care units was necessary to accommodate the broader spectrum of acute cardiovascular and non-cardiac conditions.

Today, the concept of a multidisciplinary approach is universally used with the aim to reduce morbidity and mortality associated with acute cardiovascular diseases and manage other critical conditions such as sepsis and respiratory failure.

Cardiogenic shock (CS) is a life-threatening condition with a poor prognosis, often requiring mechanical circulatory support. The challenges of cardiogenic shock, where again the multidisciplinary approach and teamwork is of a paramount importance, were addressed in three studies. One study investigated the still difficult phase of weaning patients off of mechanical support. Data from patients supported by Impella (Matassini et al.) were analyzed and predictors of successful weaning were searched for.

Left ventricular ejection fraction (LVEF) at the beginning of weaning and lactate variation within the first 12–24 h were the most accurate predictors of mortality during the weaning process. These findings underscore the importance of these two parameters in guiding clinical decisions during Impella weaning and the importance of monitoring hemodynamic and clinical parameters together in intensive care.

Another study examined the management and outcomes of patients experiencing acute myocardial infarction complicated by cardiogenic shock (AMI-CS) in low- and lower-middle-income countries (LLMICs) using data from the Ukrainian Multicentre Cardiogenic Shock Registry (Bilchenko et al.). The study underscored the necessity of effective protocols in managing cardiogenic shock in these regions. Notably, it identified several factors independently predictive of hospital mortality: left main stem occlusion, deterioration in reperfusion, Charlson Comorbidity Index >4, and cardiac arrest.

Further research compared the effects of different P2Y12 receptor antagonists on bleeding and outcomes in patients with myocardial infarction and cardiogenic shock (Kanic and Kompara). The study found that, while the combination of P2Y12 antagonists increased bleeding risk, it did not adversely affect treatment outcomes compared to individual P2Y12 agents like ticagrelor and prasugrel. This suggests that bleeding risk should be considered when choosing P2Y12 antagonists but that it may not necessarily impact overall mortality outcomes.

Prediction of Acute Organ Dysfunction in Intensive Care represents a crucial challenge in the cardiac intensive care unit. Three studies offered insights into prediction of delirium, acute kidney injury, and hospital-acquired pneumonia in intensive care.

Postoperative delirium (POD) is a common but often undiagnosed complication in cardiac surgery patients. A study involving 232 patients (Wang et al.) identified postoperative lactate levels, maximum temperature, and cardiopulmonary bypass time as independent predictors of POD. A predictive nomogram developed from these factors demonstrated excellent discriminatory power, suggesting the potential for early interventions to prevent POD in high-risk patients.

Similarly, acute kidney injury (AKI) is a frequent and serious complication after cardiac surgery. A study of 260 patients identified the fibrinogen-to-albumin ratio (FAR) as an independent predictor of AKI (Xu et al.). Although FAR significantly improved AKI prediction, its addition to clinical prediction models did not substantially enhance the area under the receiver operating characteristic curve. This finding highlights FAR's potential role in early AKI detection and prevention.

Hospital-acquired pneumonia (HAP) is another significant risk for patients admitted with acute heart failure (AHF) in intensive care units (ICUs). In a study of 638 patients (Polovina et al.), HAP occurred in 21.5%, with higher incidence in those with *de novo* AHF, severe congestion, and a history of stroke, diabetes, and chronic kidney disease. HAP was associated with longer hospital stays, increased need for inotropes and ventilatory support, and higher in-hospital mortality. These findings emphasize the need for targeted strategies to prevent HAP and manage its complications effectively.

Pharmacological interventions play a critical role in managing various cardiac conditions, including coronary artery spasm (CAS) and perioperative care in cardiac surgery. CAS, characterized by reversible vasoconstriction, can lead to fatal arrhythmias. Nondihydropyridine calcium channel blockers (CCBs), such as diltiazem, are recommended for treating and preventing CAS episodes. However, their use in patients with atrioventricular block (AV-B) is controversial. A case report (Zhang et al.) demonstrated the effective and safe use of diltiazem in a patient with CAS-induced complete AV-B, highlighting its potential benefits.

In cardiac surgery patients with severely reduced ventricular function, Levosimendan has been studied for its perioperative benefits. A retrospective study of 498 patients analyzed the impact of Levosimendan administration timing on outcomes (Schiefenhövel et al.). Patients who received prolonged preoperative Levosimendan treatment (“preconditioning”) had significantly lower in-hospital mortality, shorter duration of mechanical ventilation, and reduced need for continuous renal replacement therapy compared to those who received it intraoperatively or postoperatively. These results support the recommendation for preconditioning with Levosimendan to improve postoperative outcomes in high-risk cardiac surgery patients.

In conclusion, advancements in cardiac intensive care, understanding and managing cardiogenic shock, predicting acute organ dysfunction, and optimizing pharmacological treatments are critical to improving patient outcomes in cardiac care.

The articles included in the Research Topic “*Insights in Intensive Care Cardiovascular Medicine: 2022*” clearly highlight that continued research and implementation of evidence-based practices are essential for addressing the complexities of cardiac and non-cardiac emergencies in modern healthcare.

